# Assessment of biochemical outcomes in patients with primary aldosteronism after adrenalectomy based on CT scan diagnosis of unilateral adenoma without adrenal vein sampling

**DOI:** 10.3389/fonc.2022.944035

**Published:** 2022-11-16

**Authors:** Ming Zhang, Ge Bian, Jingyan Tian, Weijie Yang, Xiaoqing Wang, Changliang Chi

**Affiliations:** Department of Urology, The First Hospital of Jilin University, Changchun, Jilin, China

**Keywords:** primary aldosteronism, laparoscopic adrenalectomy, adrenal vein sampling, CT scan, primary aldosteronism surgical outcome

## Abstract

**Purpose:**

The purpose of this study was to assess the surgical outcomes of patients with primary aldosteronism when surgery was based only on CT finding of unilateral adenoma without adrenal vein sampling (AVS).

**Methods:**

This is a retrospective review of the records of patients who had undergone retroperitoneal laparoscopic adrenalectomy for primary aldosteronism based on CT scan finding of unilateral adenoma and had a follow-up of at least 6–12 months from January 2012 to December 2020 in a single center; decision for adrenalectomy was based on CT scan, and AVS was not used. The clinical and biochemical outcomes were accessed using the standardized primary aldosteronism surgical outcome (PASO) criteria. Patient’s demographics and preoperative factors were analyzed to assess for independent predictor of surgical success.

**Results:**

According to the PASO criteria, 172 patients finally enrolled in the training dataset, and 20 patients enrolled in the validation dataset. In the training dataset, complete clinical success was achieved in 71 patients (41.3%), partial success in 87 (50.6%), and absent success in 14 (8.1%). Biochemical outcomes showed that 151 patients (87.8%) were completely cured, 14 patients (8.1%) got a partial biochemical success, and an absent biochemical success was found in seven patients (4.1%). Multivariate logistic regression analysis showed that age, body mass index (BMI), tumor size, mean arterial pressure (MAP), and serum potassium were the most independent factors for incomplete biochemical success. Based on the results of statistical analysis, our study constructed a nomogram prognostic evaluation model for patients after unilateral primary aldosterone surgery.

**Conclusions:**

Laparoscopic adrenalectomy for patients with primary aldosteronism base on CT scan finding of a unilateral adenoma without AVS had a high rate of complete biochemical cure at 12 months. Risk factors for incomplete biochemical success include age, BMI, tumor size, MAP, and serum potassium. Our study constructed a nomogram prognostic evaluation model for patients after unilateral primary aldosterone surgery. The nomogram accurately and reliably predicted the incomplete biochemical success.

## Introduction

Primary aldosteronism (PA) is the underlying cause of secondary hypertension; accounts for ≈20% of cases are resistant hypertension, and the prevalence is estimated to be 5–10% in all patients with hypertension while increasing prevalence associated with greater severity of hypertension (1–4). A study from India in subjects with young hypertension showed an overall prevalence of PA to 17.8%, which increased with severity of hypertension (8.1% in grade 1 to 37.1% in grade 3) (5). PA can have a remarkably adverse impact on cardiovascular system both due to excessive circulating aldosterone and the subsequent hypertension, which lead to long-term fibrosis and remodeling in critical organs (6, 7). A cross-sectional study has reported that PA patients have a higher prevalence of cardiovascular complications at diagnosis than that of patients with essential hypertension matched for age, sex, and blood pressure (8).

PA mostly comprises two major types: aldosterone-producing adenoma (APA) and bilateral adrenal hyperplasia (BAH). APA can be cured by surgical intervention, whereas BAH should be medically treated with a mineralocorticoid receptor antagonist (1, 9, 10). The accurate differentiation between unilateral APA and BAH is crucial for optimal clinical management. Adrenal CT scanning and AVS are used for further characterize of either an APA or BAH (1, 9). CT is widely used, and it can provide structural information of the adrenals, and Endocrine Society guideline recommends that it should be done before AVS (1). However, CT has been reported to be unreliable for differentiation of unilateral from bilateral PA, and the sensitivity and specificity do not exceed 87 and 71%, respectively (11). AVS determines which adrenals are responsible for aldosterone excess; it remains the “gold” standard and the most accurate way to differentiate between the unilateral APA and BAH (1, 9). A recent multicenter research showed that patients diagnosed by CT have a decreased likelihood of achieving complete biochemical success compared with a diagnosis by AVS (12).

However, the evidence for the superiority of AVS over CT in terms of the surgical outcomes is limited. Although there were some limitations such as using different cutoff values and not doing dexamethasone suppression tests in all patients, the only randomized prospective clinical trial indicated that treatment of PA based on CT or AVS did not show significant differences in intensity of antihypertensive medication or clinical benefits for patients after a 1-year follow-up (13). In addition, AVS is expensive, more invasive testing, and requires considerable technical skill, which is only routinely available in some big medical centers worldwide. Its accuracy depends on a variety of factors (14). As a result, it is important to distinguish in which patients AVS can be avoided. According to the Endocrine Society guidelines (1), younger patients (age 35 years) with spontaneous hypokalemia, marked aldosterone excess, and unilateral adrenal lesions with radiological features consistent with a cortical adenoma on adrenal CT scan may not need AVS before proceeding to unilateral adrenalectomy. However, no more than 10% of patients with PA can avoid AVS according to these criteria (15). The aim of this study was to assess the surgical outcomes of patients with primary aldosteronism when surgery was based only on CT finding of unilateral adenoma without adrenal vein sampling; our study may help clinicians evaluate the benefits of surgery and inform patients on their expected postsurgical outcomes as well as identify which patients require close follow-up and reduce the financial burden on patients.

## Patients and methods

In the training dataset, the records of APA patients who underwent unilateral retroperitoneal laparoscopic adrenalectomy based on CT scan and had a follow-up of at least 6–12 months were retrospectively reviewed between January 2012 and December 2020 in the First Hospital of Jilin University.

Primary aldosteronism was diagnosed based on a history of hypertension with an increased plasma aldosterone concentration (PAC) (ng/dl)/plasma renin activity (PRA)(ng/ml/h) ratio ≥ 30, then one or more confirmatory tests (saline infusion or captopril challenge test) was done to definitively confirm the diagnosis.

In subtype testing, CT scans were performed on all PA patients to separate APA from BAH. The decision for adrenalectomy was based on CT scan finding of a unilateral low-density non-enhancing lesion (>7 mm) of less than 10 Hounsfield units with a normal contralateral gland.

The clinical and biochemical outcomes were accessed at 6–12 months post-surgery using the standardized primary aldosteronism surgical outcome (PASO) criteria (complete, partial, and absent successes of clinical biochemical outcomes) based on blood pressure, amount of antihypertensive used, agents plasma potassium and aldosterone concentrations, and plasma renin concentrations or activities (16).

Written informed consent from patients was obtained. The study was approved by the Ethics Committee of the First Hospital of Jilin University (ethics committee certificate number: 2022-507) and abides by the principles of the Declaration of Helsinki. Biochemical outcomes were chosen as primary endpoint; partial and absent successes were defined as incomplete success, patient’s preoperative factors that were analyzed were age, sex, BMI, MAP, smoking history, blood pressure, biochemical results, and tumor characteristics.

### Confirmatory test

For the confirmatory tests, antihypertensive medication was withheld or changed according to the guideline. Treatment with diuretics was withheld for at least 4 weeks. β-Blockers, angiotensin-converting enzyme inhibitors, and angiotensin-II receptor blockers were stopped for at least 2 weeks.

Captopril challenge test: Patients received 50-mg captopril orally at 8:00–9:00 am after sitting or standing for at least 1h, blood samples were drawn for the measurement of PRA and PAC at time zero and 2h after the challenge, with the patient remaining seated during this period, plasma aldosterone is normally suppressed by captopril (>30%). In patients with PA, it remains elevated and PRA remains suppressed.

Saline infusion test: Patients stayed in the recumbent position for at least 1h before and during the infusion of 2l of 0.9% saline over 4h; starting at 8:00 a.m., blood samples for PRA, PAC, cortisol, and plasma potassium were drawn at time zero and after 4h. During the test, the patients remained fasted, and blood pressure and heart rate were closely monitored, and only those with plasma aldosterone levels that > 10 ng/dl after the saline infusion were diagnosed with PA.

### Statistical analysis

All analyses were performed with SPSS (version 22.0) and R (version 4.1.3). Receiver operating characteristic curve was performed to find the best cutoff point for selected significant variables. Multivariate logistic regression was used for further investigation to find the independent factors to predicting a postoperative incomplete biochemical success; odds ratios (ORs) greater than 1 indicates an increased likelihood of incomplete biochemical success and an OR less than 1 indicates a decreased likelihood. The nomogram was plotted using the”rmda”package in R (version 4.1.3). In all analyses, *p* < 0.05 was considered significant.

### Training and validation queue

A total of 172 patients were eventually included in the training dataset between January 2012 and December 2020, and a total of 20 patients were included in the validation dataset between January and December 2021. The training cohort is used to build predictive models and stratify prognostic risks. The validation cohort conducts prognostic risk stratification and validation model.

## Results

In total, 195 patients underwent unilateral retroperitoneal adrenalectomy for APA based on CT scan between 2012 and 2020. Based on the follow-up time, 172 patients (88.2%) enrolled in the training dataset for further analysis. The cohort included 90 women (42.3%), and the mean age and BMI at the time of surgery were 52.4 ± 11.0 years and 26.3 ± 1.9 kg/m^2^, respectively; hypokalemia was present in 48.2% of patients. The baseline characteristics are reported in **Table 1**.

**Table 1:** The patient’s characteristics.

Parameter	Value
Age (years)	52.1 ± 10.9
Sex, Female/Male (%)	90/82 (52.3)
Smoking history, Yes/No (%)	49/123 (28.5)
Family history of hypertension, Yes/No (%)	52/120 (28.6)
Diabetes, Yes/No (%)	19/108 (30.2)
BMI (kg/m^2^)	26.3 ± 1.9
Duration of hypertension (months)	55 (12–144)
Preoperative antihypertensive agents (number)	2.5 ± 0.8
Systolic blood pressure (mm Hg)	178 ± 16
Diastolic blood pressure (mm Hg)	106 ± 12
Mean arterial pressure (mm Hg)	130 ± 12
Serum potassium level(mmol/L)	3.56 (1.98–4.28)
Plasma aldosterone concentration (ng/dl)	32.24 (11.88–76.53)
Plasma renin activity (ng/ml/h)	0.34 (0.2–0.7)
Tumor side, Left/Right (%)	93/79 (54.0)
Tumor size (mm)	15.2 ± 3.9

Complete clinical success was achieved in 71 patients (41.3%), partial success in 87 (50.6%), and absent success in 14 (8.1%); biochemical outcomes showed that 151 patients (87.8%) completely biochemical success, 14 patients (8.1%) got a partial biochemical success, and an absent biochemical success was found in seven patients (4.1%). In univariate analysis, age, male, BMI, tumor size, and serum potassium were the main factors related to the incomplete biochemical success after adrenalectomy (**Table 2**).

**Table 2:** Comparison of clinical data between the patients with and without complete biochemical success.

Variables	Complete (*n* = 151)	Incomplete (*n* = 21)	*P*-value
Age (years)	51.4 ± 11.1	57.4 ± 7.1	0.031
Gender, *n* (%)			0.025
Male	67 (44.4)	15 (71.4)	
Female	84 (55.6)	6 (28.6)	
Smoking history, *n* (%)			0.301
Yes	41 (27.2)	8 (38.1)	
No	110 (72.8)	13 (61.9)	
Family history of hypertension, *n* (%)			0.741
Yes	45 (29.8)	7 (33.3)	
No	106 (70.2)	14 (66.7)	
Diabetes, *n* (%)			0.364
Yes	18 (11.9)	4 (19.0)	
No	133 (88.0)	17 (81.0)	
BMI (kg/m^2^), *n* (%)	26.2 ± 1.7	27.7 ± 2.6	0.001
Duration of hypertension,	54 (12–144)	57 (18–120)	0.889
Preoperative antihypertensive agents, *n* (%)	2.5 ± 0.8	2.7 ± 1.1	0.321
Systolic blood pressure (mm Hg)	178 ± 16	179 ± 15	0.853
Diastolic blood pressure (mm Hg)	106 ± 12	108 ± 14	0.572
Mean arterial pressure (MAP)	130 ± 11.9	131 ± 11.3	0.631
Hypokalemia, *n* (%)			< 0.001
Yes	79 (51.9)	4 (19.0)	
No	62 (48.1)	17 (81.0)	
Plasma aldosterone (ng/dl)	31.83 ± 10.3	35.23 ± 10.48	0.16
Plasma renin activity (ng/ml/h)	0.3 (0.2–0.7)	0.3 (0.2–0.5)	0.3
Tumor side, *n* (%)			0.763
Left	81 (53.6)	12 (57.1)	
Right	70 (46.4)	9 (42.9)	
Tumor size (mm)	15.5 ± 3.8	13.1 ± 4.1	0.01

**Figure 1:**
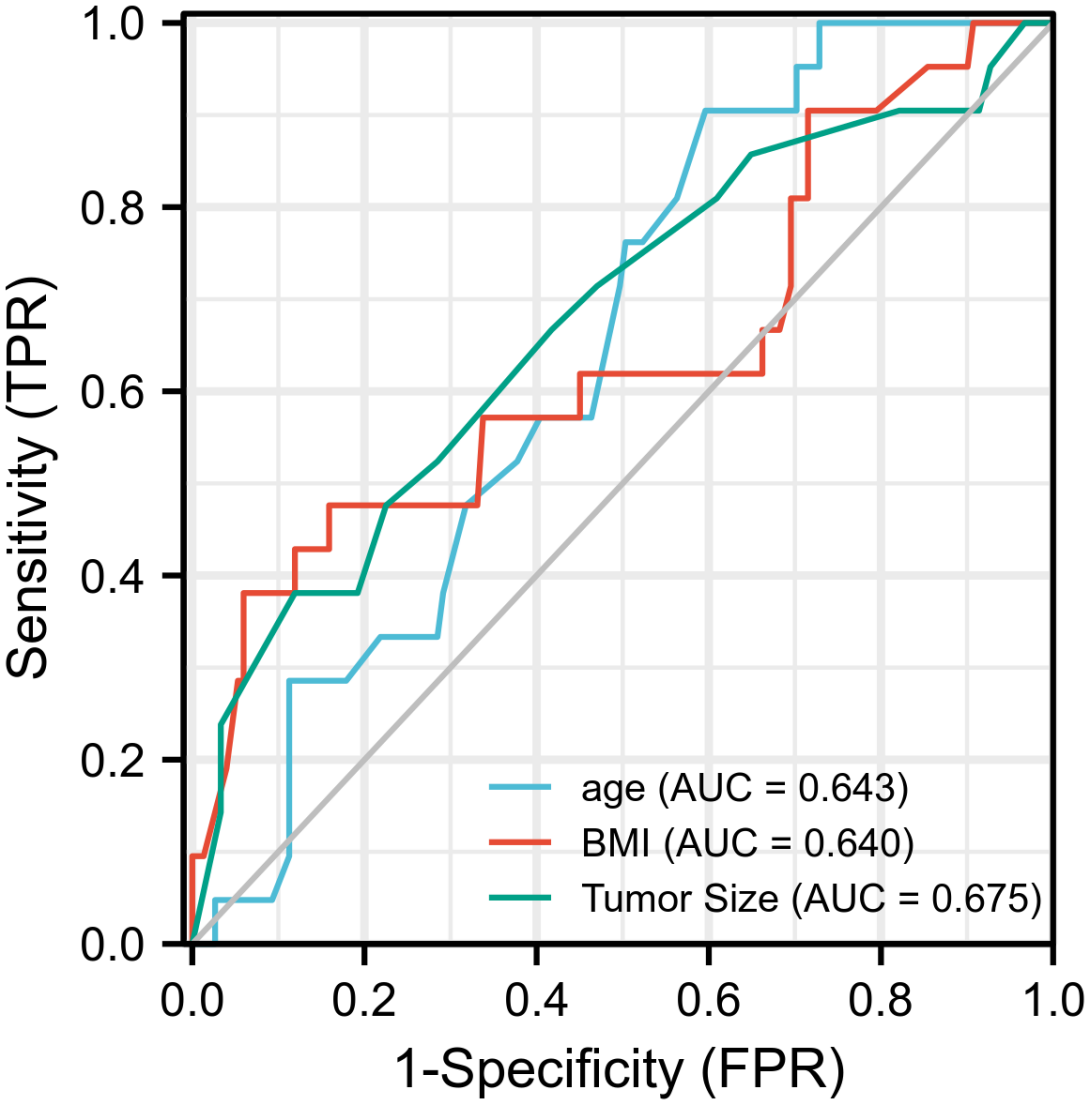
Prognostic value validation of age ≥ 50, BMI > 28.4, tumor size ≤ 10 mm by using ROC curve.

In the receiver operating characteristic curve, the best cutoff point of age was 49.5 years (area under the curve [AUC] = 0.643) for incomplete biochemical success. In BMI, the best cutoff point of weight was 28.4 kg/m^2^, respectively (AUC = 0.64), for incomplete biochemical success. The best cutoff point of tumor size was 10.5 mm, respectively (AUC = 0.67), for incomplete biochemical success (**Figure 1**). Multivariate logistic regression analysis showed that age, BMI, tumor size, MAP, and serum potassium were the most independent factors for incomplete biochemical success (**Table 3**).

**Table 3:** Predictive factors for incomplete biochemical success: Outcome of multivariate logistic regression analysis.

	B	S.E.	Wald	P	OR	95% CI
Gender (male)	0.726	0.583	1.548	0.213	2.066	0.659–6.478
BMI	0.436	0.159	7.556	0.006	1.546	1.133–2.11
MAP	0.058	0.027	4.604	0.032	1.06	1.005–1.118
Potassium	1.767	0.707	6.245	0.012	5.852	1.464–23.391
Tumor size	-0.162	0.079	4.217	0.04	0.85	0.729–0.993
Age	0.088	0.035	6.223	0.013	1.092	1.019–1.169

**Figure 2:**
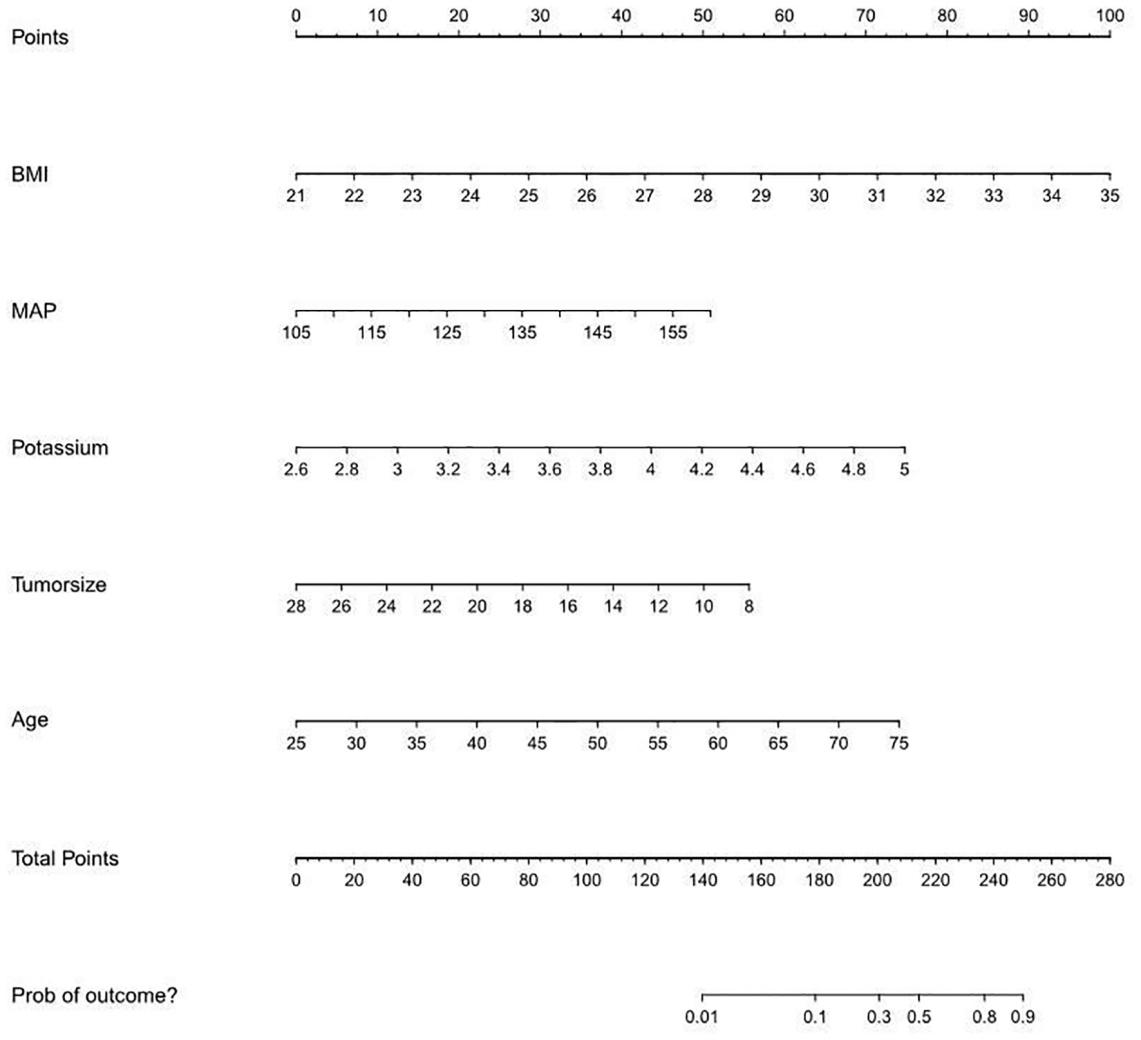
A nomogram predicting the risk of incomplete biochemical success.

**Figure 3:**
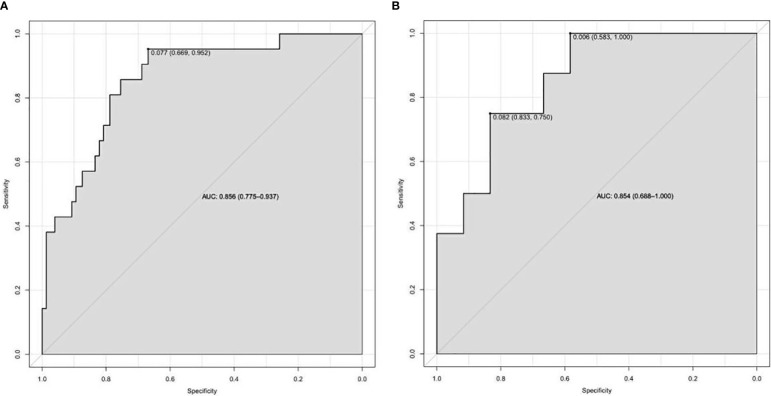
The discrimination performance of the nomogram was excellent in both the training **(A)** and validation **(B)** datasets.

**Figure 4:**
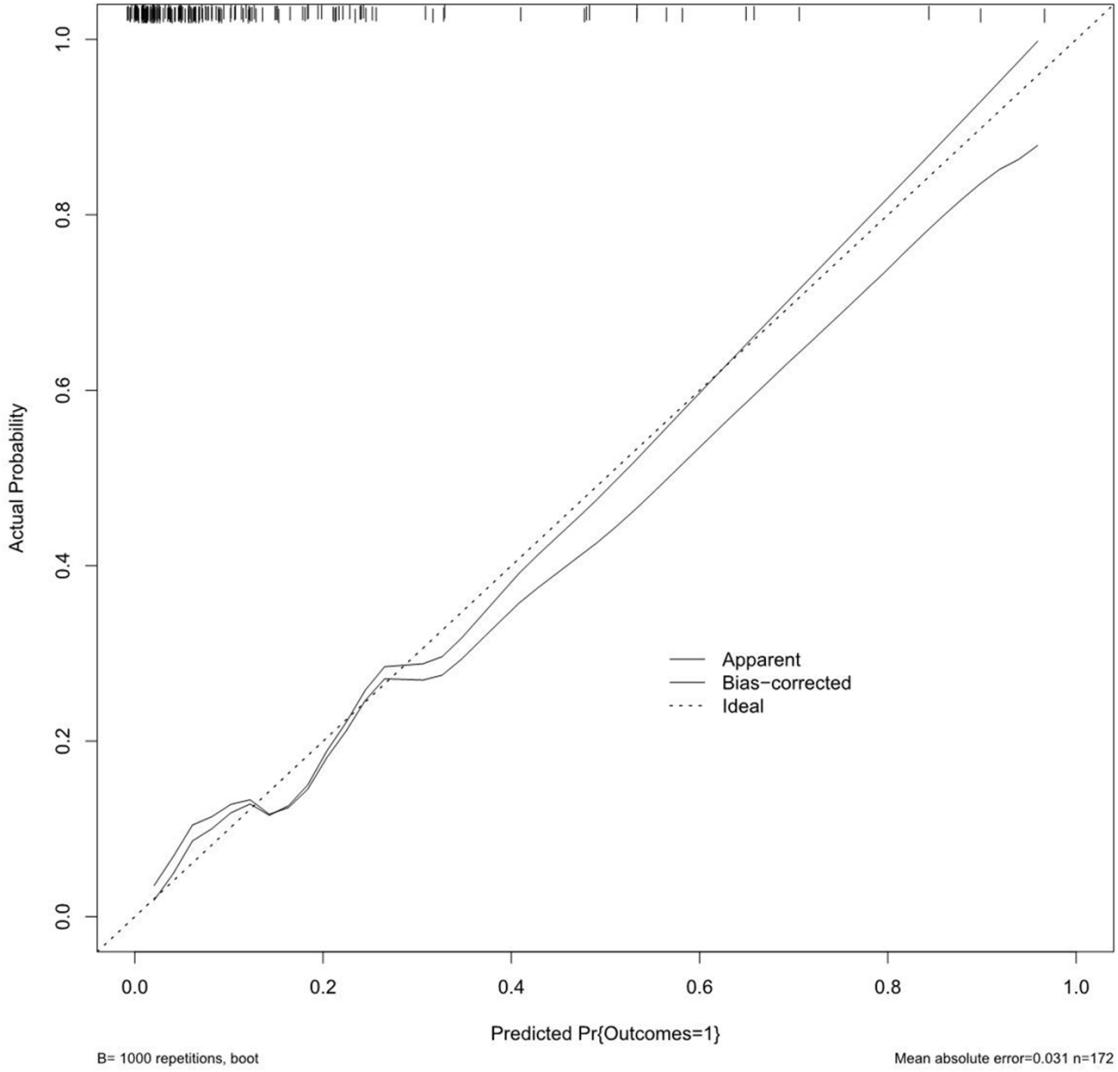
The calibration curves for the nomogram. The *x*-axis represents the nomogram-predicted probability, and *y*-axis represents the actual probability of incomplete biochemical success.

**Figure 5:**
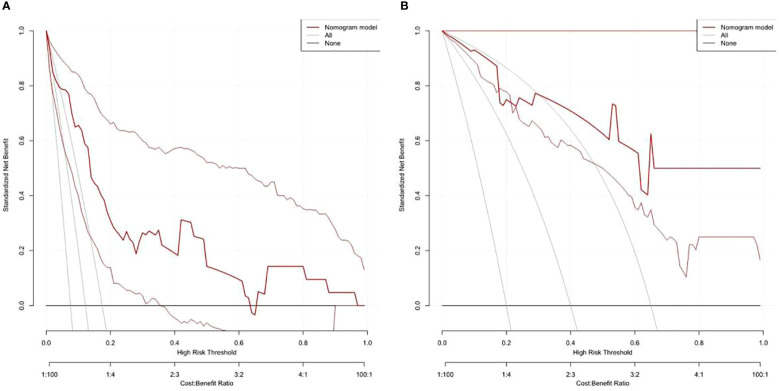
Decision curve analysis shows that the model has advantages in predicting incomplete biochemical success rates in both the training **(A)** and validation **(B)** datasets.

On the basis of the final regression analysis, a nomogram was constructed, which incorporated the five significant risk factors for predicting incomplete biochemical success (**Figure 2**). A total score was calculated using BMI, MAP, potassium, tumor size, and age; the value of each of these variables was given a score on the point scale axis; the total score could be easily calculated by adding every single score and, by projecting the total score to the lower total point scale, we were able to estimate the probability of incomplete biochemical success. The prediction model showed that older patients, higher MAP, smaller tumor size, higher BMI, and normal serum potassium had more frequent incomplete biochemical success. Based on the receiver operating characteristic analysis, the discrimination performance of the nomogram was excellent in both the training (AUC 0.856 [95% CI 0.775–0.937]) and validation (AUC 0.854 [95% CI, 0.688–1.000]) datasets (**Figure 3**). A calibration curve of the nomogram is presented in **Figure 4**. At the same time, we found that decision curve analysis (DCA) shows that the model has advantages in predicting incomplete biochemical success rates. In addition, similar DCA findings were confirmed in the verification cohort (**Figure 5**).

## Discussion

Clinically, no uniform standard exists for the cutoff of ARR for primary aldosteronism. We used the ARR cutoff of 30 ng/dl/ng/ml/h for determining screen positive PA in this study. The Endocrine Society guidelines recommend the most commonly adopted cutoff values are 30 for PAC and PRA. Meanwhile, a recent retrospective analysis found that the maximum Youden index (0.848) was obtained when the ARR cutoff value was 30 (compared with 20/40), with a sensitivity of 85%, specificity of 99.8%, negative predictive value of 96.1%, and a positive predictive value of 100% (17). Additionally, the expert consensus on the diagnosis and treatment of primary aldosteronism and the Chinese urological guidelines (18) also recommend the ARR cutoff of 30 for patients with primary aldosteronism. Therefore, the ARR cutoff of 30 ng/dl/ng/ml/h may be acceptable for determining a positive PA screen.

The accurate distinction of unilateral APA and BAH is imperative to select correctly surgical candidates with PA who can be expected to benefit from unilateral adrenalectomy. The Endocrine Society guideline recommends AVS for distinguishing APA and BAH subtypes; it is currently the “gold” standard for subtyping PA patients (1). The recent adrenal vein sampling international study showed that surgery guided by AVS provided a higher rate of cure of hypertension than when non-AVS guided (19). However, another recent international multicenter study showed that the biochemical success of unilateral PA based on AVS after surgery was 93.3%, which means that nearly 7% patients were misdiagnosed and 2% patients were potentially performed adrenalectomy on the wrong side (12). Although the above published date questioned the AVS, it remains debatable as to whether SPARTACUS has managed to definitively refute the long-held view that AVS is still the best (20). The procedure is not standardized, and lack of technical expertise medical staff may be the reasons why AVS is not available to all patients and all medical centers.

CT scan is a relatively inexpensive and non-invasive procedure, but it is generally reported to lack sensitivity for the detection of adrenal microadenomas (< 10 mm) (21–23). However, modern CT scan using thinner slices may detect microadenomas of less than 1 cm (21), and adrenal hyperplasia can be differed from adenoma by the use of CT densitometry and percentage washout (24, 25). In our cohort, partial biochemical success was achieved in 14 patients (8.1%) and absent biochemical success in seven patients (4.1%), which is broadly similar to the subgroup of CT-based patients in other studies (12). Compared with a meta-analysis of the prognosis of patients with primary aldosteronism, partial biochemical successes were similar (8.1% *vs.* 9.68%) and absent biochemical success rate was less (4.1% *vs.* 9.59%) (26); it is possible that the bias is due to the small amount of data, and the results may be close to it as the amount of data grows. Therefore, CT scan is an important method for subtyping of patients with PA in the medical centers where AVS is not routinely available. Patients with high risk of incomplete biochemical success in our model should be recommended to receive AVS; they may benefit from AVS to further reduce the rate of absent biochemical success.

Many researchers tried to find in which patients AVS can be avoided, and many clinical scores and tests were established to predict unilateral PA (27, 28). Küpers’s research showed that patients with a typical Conn’s adenoma plus serum potassium of less than 3.5 mmol/L or estimated glomerular filtration rate of at least 100 ml/min/1.73 m^2^ (or both) had a 100% specificity for unilateral primary aldosteronism diagnose (29). Kobayashi’s scoring system also showed a 100% specificity for the diagnosis of bilateral primary aldosteronism when the cutoff score is 7, and the criteria were recommended to distinguished BHA from APA in the outpatient setting before AVS (27). Jacopo Burrello’s machine learning–based models displayed an accuracy of 72.9–83.9% in confirmed diagnosis of PA (30). However, most studies used AVS not the biochemical outcomes after surgery as the reference standard. In our research, biochemical outcomes were used as the primary endpoint to find the risk factors related to incomplete biochemical success. The results showed that age, BMI, tumor size, MAP, and serum potassium were the most independent factors for incomplete biochemical success.

Our study established a nomogram prognostic evaluation model for patients after unilateral primary aldosterone surgery. A total score was calculated using BMI, MAP, potassium, tumor size, and age; the value of each of these variables was given a score on the point scale axis; the total score could be easily calculated by adding every single score; by projecting the total score to the lower total point scale, we were able to estimate the probability of incomplete biochemical success. The prediction model showed that older patients, higher MAP, smaller tumor size, higher BMI, and normal serum potassium had more frequent incomplete biochemical success. This model has great predictive power with mean absolute error = 0.031 and mean squared error = 0.00121. The discrimination performance of the nomogram was excellent in both the training (AUC 0.856 [95% CI 0.775–0.937]) and validation (AUC 0.854 [95% CI 0.688–1.000]) datasets. Therefore, the nomogram can accurately and reliably predict the incomplete biochemical success.

In our study, patients≥50 years may have an incomplete biochemical success. Patients with APA are usually believed to be younger than those with BAH (31). Recently, a study by Wachtel et al. indicated that 12% patients ≤ 40 years would have undergone unnecessary surgery, whereas 30% patients > 40 years would have undergone unnecessary surgery based on imaging (32). It has also been proposed by other research groups that patients younger than 40 years and a clearly visible unilateral adenoma in imaging can be diagnosed as APA; AVS is not necessary (33, 34). All of the above research results indicated that the BAH may occur in older patients; however, the correlation between age and BAH has not been reported.

The etiology of BAH is not yet well understood, and several studies have showed a correlation between obesity and BAH (35–37). Somlóová et al. reported that the BMI was significantly higher in BAH patients compared with that of APA patients (36). Ohno et al. reported that the prevalence of obesity was significantly higher in BAH patients than APA patients after adjusting for some clinical backgrounds, and they hypothesized that the pathogenesis of BAH may be that obesity induces the production of adipocytokines such as CTRP1, leptin, and resistin, which elevate aldosterone *via* a renin independent pathway (35). We observed that BMI was an independent risk factor–related incomplete biochemical success, which means that higher BMI may have a link with the etiology of BAH. Williams’s research also confirmed that the patients with absent clinical success had a higher BMI than that of patients with a complete or partial success (16). According to the World Health Organization criteria, BMI ≥ 30 kg/m^2^ is defined as obesity; however, we found that the best cutoff of BMI related to incomplete biochemical success was 28.4 kg/m^2^, which may due to the difference of BMIs distribution between Western countries and Asia. The criteria of weight for Chinese adults defines obesity as BMI ≥ 28 kg/m^2^ (38), and Chinese people have a higher percentage of body fat and abdominal obesity than white people at the same BMI (38, 39).

The typical radiographic characteristic of APAs is a 1.6–1.8 cm unilateral low-density non-enhancing lesion with a normal appearing contralateral adrenal gland. However, Simon et al. reported that approximately 20% of APAs will be less than 10 mm in size (40). It is difficult to distinguish between APA and BAH nodules by imaging when tumor size is less than 10 mm. In our cohort, 12.8% patients had a tumor ≤ 10 mm, and more patients had an incomplete biochemical success in this group than that of patients with a tumor > 10 mm. This may indicate that the patients with a tumor size ≤ 10 mm would have undergone unnecessary surgery based on CT scan. In addition, a substantial number of APA patients with a tumor size less than 10 mm who might be cured by surgery can be missed by CT (21). However, these patients may benefit from AVS.

Hypokalemia was reported to present in only a minority of patients with PA (9– 37%) (8). However, it occurred in 53–100% of patients with primary aldosteronism recorded in European hospital registries (41). A recent international study showed that hypokalemia was present in 73.9% unilateral PA patients (42). The Chinese urological guidelines (18) also recommend that the incidence rate of hypokalemia in primary aldosterone patients is about 50%, and a prospective study of all hypertensive inpatients (*n* = 7,594) from May 2016–April 2018 at the National Cardiovascular Center/Fu Wai Hospital in China found that 49.40% (*n* = 124) had spontaneous hypokalemia of the 251 patients with PA (43); meanwhile, another clinical research at the First Affiliated Hospital of Zhejiang University, included 222 patients diagnosed with PA from January 2009 to June 2018, found that 50% (*n* = 111) had spontaneous hypokalemia (44). It may be that the diagnostic criteria of PA varied among countries and hospital or that Asians are more sensitive to aldosterone. In our cohort, nearly 50% of the patients had a hypokalemia, and it was an independent factor related to the incomplete biochemical success. Clinically, patients with BAH are less likely to be hypokalemic compared with patients with APAs (42). In our study, half of the patients with APA had serum potassium < 3.5 mmol/L, some severe cases with serum potassium levels were below 2.0 mmol/L. Researchers has confirmed that APAs often secrete higher amounts of aldosterone, corticosterone, deoxycorticosterone, and the hybrid steroids, compared with the adrenals of BAH patients, which may be the cause of lower potassium levels in APA patients (35, 45). Thus, the presence of hypokalemia could be a high-positive predictive value for the biochemical outcomes of unilateral PA patients after adrenalectomy.

The current study has a few limitations. First, the nature of the retrospective study design is one of the weaknesses of this study, which is similar to other studies regarding PA. In addition, the small number of patients from a single center is a potential limit. Furthermore, because of the unavailability of AVS, a substantial number of patients with small tumor size or bilateral abnormalities who might be cured by surgery could be missed by CT.

## Conclusions

Although CT scan provides an anatomical and not a functional diagnosis, our results showed that laparoscopic adrenalectomy for patients with PA base on CT scan finding of a unilateral adenoma without adrenal vein sampling had a high rate of complete biochemical cure at 12 months. CT scan is an important method for subtyping of patients with PA in the medical centers where AVS is not routinely available. Our study constructed a nomogram prognostic evaluation model for patients after unilateral primary aldosterone surgery. The nomogram accurately and reliably predicted the incomplete biochemical success.

## Data availability statement

The original contributions presented in the study are included in the article/**Supplementary Material**. Further inquiries can be directed to the corresponding authors.

## Ethics statement

The studies involving human participants were reviewed and approved by the First Hospital of Jilin University. Written informed consent to participate in this study was provided by the participants’ legal guardian/next of kin.

## Author contributions

MZ and WY collected the clinical data; MZ and CC analyzed the data; JT and MZ wrote the main manuscript; GB and MZ prepared all of the tables and figures; XW and CC designed the research. All authors reviewed the manuscript. All authors contributed to the article and approved the submitted version.

## Conflict of interest

The authors declare that the research was conducted in the absence of any commercial or financial relationships that could be construed as a potential conflict of interest.

## Publisher’s note

All claims expressed in this article are solely those of the authors and do not necessarily represent those of their affiliated organizations, or those of the publisher, the editors and the reviewers. Any product that may be evaluated in this article, or claim that may be made by its manufacturer, is not guaranteed or endorsed by the publisher.
