# Supplementary material for: Assessment of biochemical outcomes in patients with primary aldosteronism after adrenalectomy based on CT scan diagnosis of unilateral adenoma without adrenal vein sampling
Source: Front Oncol. 2022 Nov 16;12:944035. doi: 10.3389/fonc.2022.944035 (PMC9710738; doi:10.3389/fonc.2022.944035)
Supplement: Supplementary file 1 [file Table_1.doc]

**International consensus on surgery outcomes for unilateral primary aldosteronism**

**Complete clinical success**

Normal blood pressure without the aid of antihypertensive

medication

**Partial clinical success**

The same blood pressure as before surgery with less

antihypertensive medication or a reduction in blood pressure

with either the same amount or less antihypertensive

medication

**Absent clinical success**

Unchanged or increased blood pressure with either the same

amount or an increase in antihypertensive medication

**Complete biochemical success**

Correction of hypokalaemia|| (if present pre-surgery) and

normalisation of the aldosterone-to-renin ratio; in patients

with a raised aldosterone-to-renin ratio post surgery,

aldosterone secretion should be suppressed in a confirmatory

test

**Partial biochemical success**

Correction of hypokalaemia (if present pre-surgery) and a

raised aldosterone-to-renin ratio with one or both of the

following (compared with pre-surgery): ≥50% decrease in

baseline plasma aldosterone concentration; or abnormal but

improved post-surgery confirmatory test result

**Absent biochemical success**

Persistent hypokalaemia|| (if present pre-surgery) or persistent

raised aldosterone-to-renin ratio, or both, with failure to

suppress aldosterone secretion with a post-surgery

confirmatory test

**Outcome assessment**

Outcome assessment should first be done in the 3 months

post surgery, but final outcome should be assessed at

6–12 months

**Annual reassessment**

Outcome should be reassessed annually

Williams TA, Lenders JWM, Mulatero P, et al. Outcomes after adrenalectomy for unilateral primary

aldosteronism: an international consensus on outcome measures and analysis of remission rates in an international cohort. Lancet Diabetes Endocrinol 2017;5:689–99.
